# Examining the influence of Covid-19 restrictions, a nurse strike, and SARS-CoV-2 coinfection on bacteremia mortality: A Danish population-based cohort study (2019–2022)

**DOI:** 10.1016/j.heliyon.2024.e33696

**Published:** 2024-06-27

**Authors:** Filip Jansåker, Mona Katrine Alberthe Holm, Jenny Dahl Knudsen, Jonas Bredtoft Boel

**Affiliations:** a Department of Clinical Microbiology, Rigshospitalet, Copenhagen, Denmark; b Center for Primary Health Care Research, Lund University, Malmö, Sweden; c Department of Clinical Microbiology, Copenhagen University Hospital Amager and Hvidovre, Copenhagen, Denmark; d Department of Clinical Microbiology, Herlev University Hospital, Herlev, Denmark; e Copenhagen University Hospital, The Hospital Pharmacy, Copenhagen, Denmark

**Keywords:** Bacteremia, Bloodstream infection, Covid-19, Mortality, SARS-CoV-2

## Abstract

**Objectives:**

Bacteremia is an acute severe infection with high mortality. Changes in healthcare services and coinfections with SARS-CoV-2 may have affected the mortality for bacteremia during the COVID-19 pandemic, which has been reported for other major diseases. In this study we examine the all-cause bacteremia mortality amidst the COVID-19 pandemic.

**Methods:**

A population-based cohort study comprised of laboratory confirmed bacteremia episodes in the Capital Region, Denmark (March 2019–February 2022). Cox proportional hazards models were used to calculate hazard ratios (HR) and 95 % confidence intervals (CI) for all-cause bacteremia mortality associated with the Covid-19 restriction period, a strike period, and coinfection with SARS-CoV-2, adjusted for possible confounders.

**Results:**

A total of 14,912 bacteremia episodes were identified in 12,693 patients during the study period. The 30- and 90-day all-cause mortality were 19 % and 27 %, respectively. The fully adjusted HR for 30- and 90-day all-cause mortality associated with the Covid-19 restriction period were 0.91 (95 % CI, 0.84 to 0.99) and 0.90 (95 % CI, 0.84 to 0.96), respectively, compared to the remaining time period. The corresponding HRs associated with SARS-CoV-2 coinfection were 1.29 (95 % CI, 1.11 to 1.50) and 1.36 (95 % CI, 1.20 to 1.55) compared to patients without coinfection. The association between the national nurse strike and all-cause bacteremia mortality was inconclusive.

**Conclusions:**

In this large population-based cohort study, a significant reduction in all-cause mortality for bacteremia was observed during the Covid-19 restriction period in Denmark, while coinfection with SARS-CoV-2 seem to be a substantial risk factor for all-cause bacteremia mortality.

## Highlights

• A Danish population-based cohort study of laboratory confirmed bacteremia.
• The 30- and 90-day all-cause mortality were 19 % and 27 %, respectively.
• The mortality was significantly lower during the Covid-19 restriction period.
• The mortality was significantly higher in those with SARS-CoV-2 coinfection.

## 1 Introduction

Bacteremia is an acute and severe infection with a large healthcare burden, comparable to myocardial infarctions and stroke [[1], [2], [3], [4]]. Short-term mortality ranges between 12 and 32 %, necessitating substantial healthcare resources for effective management of patients with bacteremia, including the imperative for early diagnosis and treatment [4,5]. These patients may therefore exhibit heightened susceptibility to even minor alterations in healthcare services [6].

The Coronavirus disease 2019 (Covid-19) pandemic, caused by the Severe acute respiratory syndrome coronavirus 2 (SARS-CoV-2) [7], has had unprecedented impact on healthcare systems worldwide [8]. Following the first confirmed Covid-19 case in Denmark (February 27, 2020) Danish authorities introduced comprehensive repercussive measurements to target the emerging pandemic [9,10]. This resulted in relatively strict policies (e.g. extensive nationwide lockdowns) and reprioritizations within the healthcare system, which occurred, with varying intensity, from March of 2020 to May of 2021 (in this paper, we refer to this period as the “Covid-19 restriction period”) [[10], [11], [12]]. During the Covid-19 restriction period, significant alterations occurred in the healthcare services, potentially resulting in collateral effects on the treatment and outcome of major diseases including bacteremia. For instance, during the initial phase of the pandemic, early reports indicated a rise in short-term mortality for major diseases in Denmark [11], including stroke [13] and myocardial infarctions [14]. However, to our knowledge, exploration of the potential consequences of healthcare changes implemented during the Covid-19 restriction period on the mortality of patients with confirmed bacteremia is lacking. With the reallocation of healthcare resources towards the management of infectious diseases, the collateral effect seen on the outcome of other acute major diseases [11,13,14] may have been different for bacteremia. Additionally, healthcare services in Denmark, were also affected by a 70-day nurse strike during the summer of 2021 [15]. As we previously identified a 5 % absolute increase in bacteremia mortality during a 60-day healthcare worker strike in 2008 [6], it is possible that bacteremia mortality was similarly impacted during the 2021 nursing strike.

Host- and microbiological factors have previously been found associated with bacteremia mortality (e.g., age, comorbidities, and polymicrobial infection) [6,16] and recently, reports of increased mortality in patients with SARS-CoV-2 infection due secondary bacteremia have emerged [[17], [18], [19]]. A substantial increase in in-hospital mortality has been reported in patients coinfected with both SARS-CoV-2 and bacteremia, surpassing the mortality observed in patients with bacteremia before the Covid-19 pandemic [18]. Therefore, it seems plausible that coinfection with SARS-CoV-2 could be a significant risk factor for all-cause mortality in bacteremia patients.

The objective of this population-based cohort study was to investigate all-cause mortality of bacteremia during the Covid-19 restriction period in Denmark, impacted by the implementation of healthcare policies in response to the Covid-19 pandemic, in comparison to the mortality of bacteremia in the adjacent period before and after. Additionally, we sought to assess the impact of a nurse strike on bacteremia mortality and examine the association between SARS-CoV-2 coinfection and all-cause mortality in bacteremia patients.

## 2 Materials and methods

### 2.1 Design and setting

This was a population-based cohort study comprising 12,693 patients with one or more laboratory confirmed bacteremia (n = 14,595) or fungemia (n = 325), in this paper cumulatively called bacteremia (n = 14,912), during study period (February 01, 2019, to May 29, 2022). The patients were residing in the Capital Region of Denmark, the largest of the five administrative regions, which provided healthcare for approximately 1.9 million (31.8 %) of the approximately 5.9 million inhabitants in Denmark during the study period. Bacteremia episodes were diagnosed at the departments of clinical microbiology (DCM) serving this population: DCM at Herlev and Gentofte University Hospital and the DCM at Amager and Hvidovre University Hospital. All Danish residents have a unique 10-digit civil registration number, which was used for linkage between data sources [20].

### 2.2 Data sources

Three data sources were used in this study. The Clinical Microbiological Laboratory Information Systems AdBact (Autonic AB, Sweden), including microbiological data such as blood cultures (bacteremia), SARS-CoV-2, and date of infection. The Danish National Patient Registry (DNPR) includes data on hospital admission and discharge diagnosis (10th International Classification of Diseases, ICD-10, codes) [21]. Because of substantial alterations in the data registration process within DNRP in January 2019, we chose to commence the study period in February 2019 to ensure the accuracy of the admissions recorded in this registry. The Danish Civil Registration System comprises information on age, sex, vital status (deceased or alive), and emigration/migration of all Danish residents, with less than 0.3 % lost to follow-up [20]. All data had been electronically recorded prospectively allowing longitudinal surveillance of the study population at the individual level.

### 2.3 Study population

The study population comprised clinically significant bacteremia episodes identified from a geographically well-defined background population between February 01, 2019, and February 28, 2022. Bacteremia episodes were identified through blood cultures, obtained upon clinical suspicion of bloodstream infection, with ≥30 mL blood. These cultures were incubated using either BACTEC or BacT/ALERT [22]. Bacteremia was defined according to globally established criteria [23]. Blood cultures with significant growth of pathogenic bacteria were considered as episodes of bacteremia, while contaminant (e.g., *Cutibacterium acnes*, coagulase-negative staphylococci, Corynebacterium spp., Bacillus spp.) were not, except if the same species were identified in a different set blood cultures from the same patient within five days, then the findings were considered significant and registered as a bacteremia episode. If growth of more than one pathogenic species were identified in the same blood culture or in other blood cultures obtained from the same day or the day after, findings were considered a polymicrobial infection. Patients could be included in the study more than once if they had multiple bacteremia episodes; a new episode was defined as a different bacterial species identified in a blood culture collected more than one day after the previous blood culture. Blood cultures displaying the same species within 30 days were, however, considered part of the same bacteremia episode. In total, the study population included 14,912 bacteremia episodes from 12,693 individuals. Of the included individuals, 11,067 (87.2 %) had one bacteremia episode, 1258 (9.9 %) had two episodes, 243 (1.9 %) had three episodes, 82 (0.6 %) had four episodes, and 43 (0.3 %) had more than four episodes during the study period. A total of 325 (2.2 %) of all 14,912 episodes were fungemia (*Candida* spp.).

### 2.4 Predictors

*COVD-19 restriction period:* The primary predictor variable was defined as the COVID-19 restriction period (March 12, 2020, to May 05, 2021) which occurred in Denmark as a response to the Covid-19 pandemic. During this period, the Danish authorities implemented stringent policies, including extensive nationwide lockdowns and restrictions [10]. Reprioritizations within the healthcare system included a shift of healthcare resources towards the management of infectious diseases.

*Strike period:* A secondary predictor variable was the nurse strike that commenced on June 19, 2021, and ended on August 28, 2021. The strike had significant implications on healthcare, particularly for planned medical activities, while patients with acute or life-threatening conditions received treatment in accordance with established guidelines [15].

*SARS-CoV-2 coinfection:* The third predictor variable was coinfection with SARS-CoV-2, which was defined as laboratory confirmed SARS-CoV-2 ± 30 days from the bacteremia episode. Detection of SARS-CoV-2 was performed through validated diagnostic tools and methods constantly evolving during the study period. This included Reverse transcription polymerase chain reaction (RT-PCR), antigen testing, and whole genome sequencing [[24], [25], [26]].

### 2.5 Outcome

Three specific outcomes were defined as 30-day, 31–90-day, and 90-day all-cause mortality. Data on all-cause mortality was collected from the Danish Civil Registration System from the start of the study period until May 28, 2022.

### 2.6 Covariates

Analyses were adjusted for Charlson Comorbidity Index score (CCI) [27] and other potential confounders (e.g., age, sex, and polymicrobial bacteremia) [6,16,28]. Variables included in the adjustments as potential confounding variables were: Month of the bacteremia episode, age, sex (male or female), acquisition of bacteremia (community acquired, hospital associated, or nosocomial), mono- or polymicrobial bacteremia, and CCI at baseline (categorized as low [0 points], medium [1–2 points] or high [>2 points] CCI score) [6,16,27,28]. Community acquired bacteremia was defined as no hospital contact within 30 days prior to the bacteremia episode, hospital associated bacteremia was defined as bacteremia occurring within 30 days after hospital contact/discharge (excluding the first two days after discharge), and nosocomial bacteremia was defined as bacteremia occurring between two days after hospital admission until two days after discharge [29]. Mono- and polymicrobial bacteremia was defined as significant growth of one or more than one pathogenic bacteria in the blood cultures, respectively. CCI was calculated at baseline using on ICD-10 code for CCI conditions identified within ten years preceding baseline.

### 2.7 Statistical analyses

Baseline was set to the time of bacteremia diagnosis during the time period (February 01, 2019, to February 28, 2022). Patients were followed in terms of all-cause mortality, 90 days following the bacteremia episode, with censoring for emigration, until end of follow-up (May 29, 2022), whichever came first. Descriptive statistics were presented on all variables. Cox proportional hazards models were used to estimate the association between the predictor variables and outcomes. The results were presented as hazard ratios with 95 % confidence intervals (CI). Mortality among patients with bacteremia during the Covid-19 restriction period was compared to the mortality during the remaining study period. The mortality during the strike period was likewise compared to the mortality during the remaining study period. In addition, the mortality in patients with SARS-CoV-2 coinfection was compared to those without SARS-CoV-2 coinfections.

Analyses were conducted in three models: Model 1 was an age adjusted model for each predictor (Covid-19 restriction period, coinfections with SARS-CoV-2 and strike period); Model 2 was adjusted for age and the predictor variables; and Model 3 was a full model, adjusted for age, the predictor variables, and covariates. To accommodate that some patients could have had multiple bacteremia episodes, episodes were clustered within individual patients. We tested the model for proportionality using Schoenfeld residuals (which was fulfilled). We also performed a time-split analysis (for day 0–30 and day 31–90) to identify any difference in mortality during the 90-day follow-up. A sensitivity analysis was conducted including only the first bacteremia episode from each patient during the study period. All analyses were performed in R version 4.3.0.

## 3 Results

We identified 14,912 bacteremia episodes in 12,693 patients during the study period. The 30-day and 90-day all-cause mortality were 19 % (2905 patients) and 27 % (4092 patients), respectively.

Table 1 includes characteristics and 90-day all-cause mortality for the 14,912 bacteremia episodes. The 90-day mortality was 25 % during the nurse strike, 27 % during the Covid-19 restriction period, and 41 % in patients with SARS-CoV-2 coinfection. Table S1 displays similar characteristics but slightly lower mortality, including only first episode of bacteremia during the study period for each patient.

**Table 1:** Study population characteristics and 90-day mortality in patients with bacteremia.

Characteristic	Study population^1^ n = 14,912	Dead at day 90 n = 4092	Alive at day 90 n = 10,820
**Sex**			
Female	6405 (100 %)	1677 (26 %)	4728 (74 %)
Male	8507 (100 %)	2415 (28 %)	6092 (72 %)
**Age**^**2**^	75 (65, 83)	79 (71, 86)	74 (62, 81)
**Origin of infection**			
Community acquired	7536 (100 %)	1483 (20 %)	6053 (80 %)
Healthcare associated	3571 (100 %)	1128 (32 %)	2443 (68 %)
Nosocomial	3805 (100 %)	1481 (39 %)	2324 (61 %)
**Charlson Comorbidity Index**			
Low (0 points)	3826 (100 %)	577 (15 %)	3249 (85 %)
Medium (1–2 points)	5413 (100 %)	1517 (28 %)	3896 (72 %)
High (>2 points)	5673 (100 %)	1998 (35 %)	3675 (65 %)
**Pathogen**			
*Escherichia coli*	4777 (100 %)	945 (20 %)	3832 (80 %)
*Enterococcus faecalis*	396 (100 %)	118 (30 %)	278 (70 %)
*Enterococcus faecium*	515 (100 %)	259 (50 %)	256 (50 %)
*Klebsiella pneumoniae*	951 (100 %)	262 (28 %)	689 (72 %)
*Pseudomonas aeruginosa*	263 (100 %)	95 (36 %)	168 (64 %)
*Staphylococcus aureus*	1867 (100 %)	615 (33 %)	1252 (67 %)
*Streptococcus pneumoniae*	340 (100 %)	74 (22 %)	266 (78 %)
*Streptococcus pyogenes*	113 (100 %)	27 (24 %)	86 (76 %)
Coagulase-negative staphylococci	260 (100 %)	84 (32 %)	176 (68 %)
*Candida* species	325 (100 %)	187 (58 %)	138 (42 %)
Polymicrobial	1349 (100 %)	484 (36 %)	865 (64 %)
Other species	3756 (100 %)	942 (25 %)	2814 (75 %)
**Time period of infection**			
February 01, 2019, to March 11, 2020	5359 (100 %)	1467 (27 %)	3892 (73 %)
Covid-19 restriction period (March 12, 2020, to May 20, 2021)	5655 (100 %)	1516 (27 %)	4139 (73 %)
May 21, 2021, to June 18, 2021	361 (100 %)	94 (26 %)	267 (74 %)
Strike period (June 19, 2021, to August 28, 2021)	975 (100 %)	245 (25 %)	730 (75 %)
August 29, 2021, to February 28, 2022	2562 (100 %)	770 (30 %)	1792 (70 %)
**Coinfection**^**3**^**with SARS-CoV-2**	746^4^ (100 %)	307 (41 %)	439 (59 %)

(1) Number of bacteremia episodes (%) between February 01, 2019, to February 29, 2022, and mortality follow-up until May 29, 2022. (2) Median (inter quartile range). (3) Laboratory confirmed Covid-19 infection within ± 30 days from the bacteremia episode. (4) Corresponding to SARS-CoV-2 co-infection in approximately 8.4 % of all bacteremia episodes diagnosed between March 2020 (following the first confirmed Covid-19 case in Denmark on February 28, 2020) and February 2022.

Table 2 shows the results from the Cox regression analysis on all-cause mortality for bacteremia (14,912 episodes). The results indicate lower mortality during the Covid-19 restriction period, compared to that of the remaining period. The fully adjusted HRs for 30- and 90-day all-cause mortality for bacteremia associated with the Covid-19 restriction period were 0.91 (95 % CI, 0.84 to 0.99) and 0.90 (95 % CI, 0.84 to 0.96), respectively, compared to the remaining period. Findings were similar in the sensitivity analysis, which included only the first bacteremia episode for each patient during the study period (Table S2); e.g., the HR for 90-day all-cause mortality associated with the Covid-19 restriction period was 0.91 (95 % CI, 0.85 to 0.99) compared to the remaining study period.

**Table 2:** Associations between Covid-19 restriction period, coinfection with SARS-CoV-2, strike period, and all-cause mortality in patients with bacteremia (14,912 cases).

Predictor variables	All-cause mortality	Model 1		Model 2		Model 3	
		HR (95 % CI)	p-value	HR (95 % CI)	p-value	HR (95 % CI)	p-value
**Covid-19 restriction period**^**1**^ (ref. remaining period)	0–30 days	0.96 (0.89–1.04)	0.33	0.93 (0.86–1.01)	0.089	0.91 (0.84–0.99)	0.023
	31–90 days	0.89 (0.79–1.01)	0.075	0.89 (0.79–1.02)	0.086	0.87 (0.76–0.99)	0.037
	0–90 days	0.94 (0.88–1.01)	0.082	0.92 (0.86–0.99)	0.021	0.90 (0.84–0.96)	0.003
**Strike period**^**2**^ (ref. remaining period)	0–30 days	0.80 (0.68–0.95)	0.009	0.80 (0.67–0.94)	0.009	0.90 (0.75–1.09)	0.28
	31–90 days	1.11 (0.88–1.40)	0.39	1.09 (0.86–1.38)	0.50	1.22 (0.95–1.56)	0.13
	0–90 days	0.89 (0.78–1.02)	0.10	0.88 (0.77–1.01)	0.078	1.00 (0.85–1.17)	0.96
**Coinfection**^**3**^**with SARS-CoV-2** (ref. no coinfection)	0–30 days	1.60 (1.38–1.85)	<0.001	1.59 (1.37–1.84)	<0.001	1.29 (1.11–1.50)	<0.001
	31–90 days	1.86 (1.46–2.37)	<0.001	1.88 (1.47–2.39)	<0.001	1.56 (1.22–1.99)	<0.001
	0–90 days	1.66 (1.47–1.89)	<0.001	1.66 (1.47–1.89)	<0.001	1.36 (1.20–1.55)	<0.001

HR = Hazard ratio; CI = Confidence interval. Model 1: Age adjusted HR for each predictor variable. Model 2: Adjusted for the predictor variables and age. Model 3: Full model adjusted for the predictor variables and all covariates (age, sex, month of bacteremia, acquisition of bacteremia [community acquired, hospital associated, or nosocomial], pathogen, and Charlson comorbidity index [categorized as low, medium or high index score]). (1) March 12, 2020, to May 20, 2021. (2) June 19, 2021, to August 28, 2021. Study period: February 01, 2019, to February 28, 2022, with follow-up for mortality until May 29, 2022. (3) Laboratory confirmed Covid-19 infection within ± 30 days from the bacteremia episode.

Findings regarding the correlation between the strike period and all-cause mortality for bacteremia yielded inconclusive results. Nonetheless, the results suggest that the 30-day all-cause mortality for bacteremia might have been lower during the strike period, i.e., the age-adjusted hazard ratio (HR) for 30-day all-cause mortality associated with the strike period was 0.80 (95 % CI, 0.68 to 0.95) compared to the remaining period. The association disappeared in the full model and the sensitivity analysis showed no significant associations.

SARS-CoV-2 coinfection was strongly associated with all-cause mortality for bacteremia. The age-adjusted HR for 30-day and 90-day all-cause mortality associated with SARS-CoV-2 coinfections was 1.60 (95 % CI, 1.38 to 1.85) and 1.66 (95 % CI, 1.47 to 1.89), respectively, compared to those without SARS-CoV-2 coinfection (Table 2). The associations remained in Model 2 but were somewhat attenuated in the full model. The fully adjusted HRs for 30- and 90-day all-cause bacteremia mortality associated with SARS-CoV-2 coinfection were 1.29 (95 % CI, 1.11 to 1.50) and 1.36 (95 % CI, 1.20 to 1.55) compared to no coinfection, respectively. The findings were similar in the sensitivity analysis (Table S2).

We also conducted an additional fully adjusted sensitivity analysis on 90-day mortality associated with SARS-CoV-2 coinfection in the Covid-19 restriction period and the remaining period (data not shown). This analysis yielded a HR for 90-day mortality associated with SARS-CoV-2 of 1.53 (95 % CI, 1.28 to 1.83; p < 00.001) compared to no SARS-CoV-2 coinfection during the Covid-19 restriction period. The corresponding HR associated with SARS-CoV-2 coinfection was 1.22 (1.02–1.46, p = 0.027) during the remaining period. We found no significant difference between SARS-CoV-2 coinfection during restriction period and the remaining period (p = 0.079).

## 4 Discussion

This population-based study examined all-cause mortality in patients with laboratory confirmed bacteremia during the Covid-19 pandemic and adjacent time periods in Denmark. We observed an approximately 10 % reduction in 30- and 90-day all-cause mortality for bacteremia during the Covid-19 restriction period in Denmark. Coinfection with SARS-CoV-2 was associated with approximately 29 % and 36 % increased 30- and 90-day mortality, respectively. The findings suggest lower all-cause mortality following a bacteremia episode during the Covid-19 restriction period, whereas coinfection with SARS-CoV-2 was independently associated with increased bacteremia mortality. The findings regarding the nurse strike and bacteremia mortality were inconclusive.

The decrease in all-cause bacteremia mortality during the Covid-19 restrictions period contrasts with previous findings of increases in short-term mortality in other major diseases in Denmark [11,13,14]. One explanations for this could be, as previously suggested [11,13,14], that while the management and healthcare for major (non-infectious) diseases such as stroke [13] and myocardial infarctions [14] were negatively affected by the reprioritizations in the healthcare system during the Covid-19 restrictions period, the opposite is possible for bacteremia. The heightened emphasis on infectious diseases care during the Covid-19 restriction period could potentially have had a beneficial spillover impact on the management of bacteremia. In addition, patients with symptoms of bacteremia may have been diagnosed and treated faster during the Covid-19 restriction period, due to clinical presentation [30] overlapping with that of Covid-19 [31].

The finding that coinfection with SARS-CoV-2 was associated with higher bacteremia mortality was expected. Previous studies have reported higher short-term mortality in patients infected with SARS-CoV-2 who acquire secondary bacteremia [[17], [18], [19]]. Polymicrobial infection is an established risk factor for increased bacteremia mortality, independent of other risk factors such as age, sex, and comorbidities (CCI) [28]. Similarly, our results suggest that coinfection with SARS-CoV-2 is an independent risk factor for increased bacteremia mortality. The mechanism behind this could be interactions between virus and bacteria that promote the infection process [32], which could be supported by the cumulative evidence that the mortality in viral pandemics seem to have been significantly impacted by bacterial coinfections [[32], [33], [34]].

The impact of the 2021 nurse strike on bacteremia mortality was inconclusive, though there was a slight indication of reduced mortality during the strike period. This contrasts with our previous findings, of a 5 % absolute increase in bacteremia mortality during a healthcare worker strike in 2008 [6]. The observed inconsistency could be attributed to various factors, including potentially lower stress on the healthcare system caused by the 2021 strike compared to the 2008 strike. This variation could be a result of heightened precautions in the management of acute diseases and an increased emphasis on managing infectious diseases, influenced by the Covid-19 pandemic [9,10,12].

Important limitations of this study are the absence of data regarding symptoms as well as the severity of the bacteremia. We also did not have data on given empirical antibiotic treatments; however, the empirical antibiotic treatment recommendations were ampicillin and gentamicin (with/without metronidazole) or piperacillin-tazobactam throughout the study period. Moreover, although we adjusted for important potential confounders the possibility of residual confounding cannot be ruled out, and a causal effect cannot be attributed to our findings. Another limitation is that the 2021 nurse strike occurred with varying intensity in Denmark, with the Capital Region being less affected than the North Jutland Region [15]. This aspect should be considered when interpreting our findings. However, our study has several strengths that balances out the limitations. Firstly, our study relied on validated microbiological laboratory data and comprehensive registers [20,21]. Secondly, we have previously conducted a similar study on a healthcare worker strike in 2008 in Denmark [6] and were able to investigate the impact of a recent strike on bacteremia mortality in the same country. Moreover, all coinfections with SARS-CoV-2 were based on microbiological laboratory findings using validated diagnostic tools and methods, such as RT-PCR [[24], [25], [26]], and did not include infections diagnosed by self-testing, which could have been more prone to misdiagnosis [35]. Lastly, the observed all-cause mortality patterns in relation to month of the bacteremia episode, sex, acquisition form of bacteremia, mono- or polymicrobial bacteremia, and CCI index [27] were expected [6,16,28]. This consistent pattern reinforces the reliability and validity of our data and strengthens the overall robustness of our findings.

Our findings have several clinical and public health implications. The Covid-19 pandemic prompted governments worldwide to implement various policies and engage in resource reprioritization [8,10], resulting in various collateral effects on society and healthcare [11,[36], [37], [38]]. These effects included an elevated mortality for acute major diseases such as stroke and myocardial infarction [11,13,14]. However, our study demonstrates that these collateral effects are not consistent for all acute major diseases. Conversely, our findings suggest that the reprioritization of healthcare resources to the management of infectious diseases had a favorable prognostic impact on bacteremia. Nevertheless, our study also highlights that coinfection with SARS-CoV-2 was associated with higher mortality in patients with bacteremia. Similar observations have been reported previously [[17], [18], [19]] regarding secondary bacteremia in patients with SARS-CoV-2 as well as in past viral pandemics [[32], [33], [34]]. While our study indicates that policies related to the Covid-19 pandemic appear to have improved the prognosis for bacteremia, it also emphasizes the necessity of integrating prevention and early treatment of bacterial coinfections in future pandemic planning [33]. Clinicians treating patients with bacteremia should be aware of the increased mortality associated with viral coinfections, particularly considering that approximately 8.4 % of all bacteremia episodes between March 2020 (following the first confirmed Covid-19 case in Denmark on February 28, 2020) and February 2022 in our study had coinfections with SARS-CoV-2, underscoring the need for extra precautions and care for patients diagnosed with bacteremia in future viral outbreaks and pandemics.

Further research in other settings (e.g., in countries with different policies during the Covid-19 pandemic [10]) is needed to identify the specific mechanisms that may have contributed to the observed inconsistencies regarding collateral effects on the prognosis of acute major diseases. Similar studies on less severe but common conditions in primary care are also needed. Moreover, it is also important to continue evaluating potential negative effects of healthcare worker strikes [6], to help identify areas where increased precautions are needed in future strikes, which can save lives.

In conclusion, our study revealed a notable reduction in all-cause mortality of bacteremia during the Covid-19 restriction period in Denmark. However, it is important to note that coinfection with SARS-CoV-2 appears to be a considerable risk factor for all-cause mortality in patients with bacteremia. Although the study cannot prove causality, it is plausible that the reprioritizations within the healthcare system during the Covid-19 restriction period had a positive effect on the management of bacteremia. Our findings indicate that not all major diseases were negatively impacted by the collateral effects attributed to the Covid-19 restrictions in Denmark.

**Ethics statement:** This population-based study was approved according to the guidelines of the Regional Committee on Health Research Ethics for use of clinical and laboratory data (Danish Data Protection Agency, record R-22036283). All data were pseudonymized for the researchers. As a registry study utilizing previously collected secondary data, obtaining informed consent from individuals was neither possible nor required.

## Funding

Filip Jansåker wishes to thank Region Skåne and Lund University for supporting this research by Swedish govermental funding of clinical research(ALF 2023-YF0030); and the Swedish Society of Medicine (SLS-960562, SLS-960574), Tore Nilsons Stiftelse För Medicinsk Forskning (2022-006, 2023-073), the SSAC Foundation (SLS-986553), the Crafoord Foundation (20230633), Thelma Zoégas Foundation (TZ2021-0003), the Royal Physiographic Society of Lund (42795), and Anna-Britt & Bengt Attefelts foundation (221012) for supporting this research. The funding sources of the study were all non-commercial and had no role in the study design; the collection, analysis, and interpretation of data; the writing of the report; or in the decision to submit the paper for publication.

## 5 Data availability statement

This study utilized existing secondary data gathered from population-based registers and laboratory databases. Due to legal restrictions, the data cannot be publicly shared. The data analysis code can be provided upon reasonable request to the corresponding author of this study.

## CRediT authorship contribution statement

**Filip Jansåker:** Writing – original draft, Visualization, Validation, Supervision, Software, Resources, Project administration, Methodology, Investigation, Funding acquisition, Conceptualization. **Mona Katrine Alberthe Holm:** Writing – review & editing, Visualization, Validation, Methodology, Investigation, Conceptualization. **Jenny Dahl Knudsen:** Writing – review & editing, Visualization, Validation, Supervision, Resources, Project administration, Methodology, Investigation, Data curation, Conceptualization. **Jonas Bredtoft Boel:** Writing – review & editing, Visualization, Validation, Supervision, Software, Resources, Project administration, Methodology, Investigation, Formal analysis, Data curation, Conceptualization.

## Declaration of Competing interest

All authors affirm that this manuscript is conducted honest, accurate, and transparent. All authors have completed the author submission statement. All authors declare no support from any organization for the submitted work; no financial relationships with any organizations that might have an interest in the submitted work in the previous three years, no other relationships or activities that could appear to have influenced the submitted work.

## References

[1] Goto M., Al-Hasan M.N. (2013). Overall burden of bloodstream infection and nosocomial bloodstream infection in North America and Europe. Clin. Microbiol. Infect..

[2] Holm M.K.A. (2021). Decrease in all-cause 30-day mortality after Bacteraemia over a 15-year period: a population-based cohort study in Denmark in 2000-2014. Int. J. Environ. Res. Publ. Health.

[3] Laupland K.B. (2013). Incidence of bloodstream infection: a review of population-based studies. Clin. Microbiol. Infect..

[4] Schechner V. (2022). One-year mortality and years of potential life lost following bloodstream infection among adults: a nation-wide population based study. Lancet Reg Health Eur.

[5] Paul M., Greub G. (2015). The hidden killer: are we improving the management of bacteremia?. Clin. Microbiol. Infect..

[6] Jansaker F. (2021). All-cause mortality due to bacteremia during a 60-day non-Physician healthcare worker strike. Clin. Infect. Dis..

[7] Dhama K. (2020). Coronavirus disease 2019-COVID-19. Clin. Microbiol. Rev..

[8] Hale T. (2021). A global panel database of pandemic policies (Oxford COVID-19 Government Response Tracker). Nat. Human Behav..

[9] Holler J.G. (2021). First wave of COVID-19 hospital admissions in Denmark: a Nationwide population-based cohort study. BMC Infect. Dis..

[10] Mishra S. (2021). Comparing the responses of the UK, Sweden and Denmark to COVID-19 using counterfactual modelling. Sci. Rep..

[11] Bodilsen J. (2021). Hospital admission and mortality rates for non-covid diseases in Denmark during covid-19 pandemic: nationwide population based cohort study. BMJ.

[12] Ssi, Statens Serum Institut, Denmark (februar 2022). Corona Restrictions during Covid-19-Epidemic (February 2020 to March 2022); in Danish: Coronarestriktioner under Covid-19-Epidemien.

[13] Simonsen C.Z. (2022). COVID-19 did not result in increased hospitalization for stroke and transient ischemic attack: a nationwide study. Eur. J. Neurol..

[14] De Luca G. (2022). COVID-19 pandemic, mechanical reperfusion and 30-day mortality in ST elevation myocardial infarction. Heart.

[15] The National Board of Health in Denmark (Danish: Sundhedsstyrelsen) (2021). “It Will Take Time to Get the Backlog after the Nursing Strike in the Hospital System” (Danish: Det Vil Tage Tid at Få Afviklet Efterslæbet Efter Sygeplejerskestrejken I Sygehusvæsenet).

[16] Holmbom M. (2016). 14-Year Survey in a Swedish county Reveals a Pronounced increase in bloodstream infections (BSI). Comorbidity - an independent risk factor for both BSI and mortality. PLoS One.

[17] Nakagawara K. (2023). Diagnostic significance of secondary bacteremia in patients with COVID-19. J. Infect. Chemother..

[18] Patton M.J. (2023). COVID-19 bacteremic co-infection is a major risk factor for mortality, ICU admission, and mechanical ventilation. Crit. Care.

[19] Santos C.V. (2022). Incidence of bloodstream infections in patients with COVID-19: a retrospective cohort study of risk factors and outcomes. Germs.

[20] Schmidt M., Pedersen L., Sorensen H.T. (2014). The Danish civil registration system as a tool in epidemiology. Eur. J. Epidemiol..

[21] Schmidt M. (2015). The Danish National Patient Registry: a review of content, data quality, and research potential. Clin. Epidemiol..

[22] Menchinelli G. (2019). In vitro evaluation of BACT/ALERT(R) VIRTUO(R), BACT/ALERT 3D(R), and BACTEC FX Automated blood culture systems for detection of microbial pathogens using Simulated Human blood Samples. Front. Microbiol..

[23] Gradel K.O. (2014). The Danish Collaborative Bacteraemia Network (DACOBAN) database. Clin. Epidemiol..

[24] Fomsgaard A.S., Rosenstierne M.W. (2020). An alternative workflow for molecular detection of SARS-CoV-2 - escape from the NA extraction kit-shortage, Copenhagen, Denmark, March 2020. Euro Surveill..

[25] Rosenstierne M.W. (2021). SARS-CoV-2 detection using reverse transcription strand invasion based amplification and a portable compact size instrument. Sci. Rep..

[26] Milbak J. (2021). A prospective cohort study of confirmed severe acute respiratory syndrome coronavirus 2 (SARS-CoV-2) infection during pregnancy evaluating SARS-CoV-2 antibodies in maternal and umbilical cord blood and SARS-CoV-2 in vaginal swabs. Acta Obstet. Gynecol. Scand..

[27] Charlson M.E. (1987). A new method of classifying prognostic comorbidity in longitudinal studies: development and validation. J. Chron. Dis..

[28] Sogaard M. (2011). Blood culture status and mortality among patients with suspected community-acquired bacteremia: a population-based cohort study. BMC Infect. Dis..

[29] Haque M. (2018). Health care-associated infections - an overview. Infect. Drug Resist..

[30] Rector W.G. (1981). Fever, shock and chills in gram-negative bacillemia: clinical correlations in 100 cases. Johns Hopkins Med. J..

[31] da Rosa Mesquita R. (2021). Clinical manifestations of COVID-19 in the general population: systematic review. Wien Klin. Wochenschr..

[32] Almand E.A., Moore M.D., Jaykus L.A. (2017). Virus-bacteria interactions: an emerging Topic in Human infection. Viruses.

[33] MacIntyre C.R. (2018). The role of pneumonia and secondary bacterial infection in fatal and serious outcomes of pandemic influenza a(H1N1)pdm09. BMC Infect. Dis..

[34] Morris D.E., Cleary D.W., Clarke S.C. (2017). Secondary bacterial infections associated with influenza pandemics. Front. Microbiol..

[35] Keaney D. (2021). Misdiagnosis of SARS-CoV-2: a Critical review of the influence of Sampling and clinical detection methods. Med. Sci..

[36] Mansfield K.E. (2021). Indirect acute effects of the COVID-19 pandemic on physical and mental health in the UK: a population-based study. Lancet Digit Health.

[37] Muniz-Pardos B. (2020). Collateral health Issues Derived from the covid-19 pandemic. Sports Med Open.

[38] Xiao H. (2021). The impact of the COVID-19 pandemic on health services utilization in China: time-series analyses for 2016-2020. Lancet Reg Health West Pac.

