# Supplementary material for: Examining the influence of Covid-19 restrictions, a nurse strike, and SARS-CoV-2 coinfection on bacteremia mortality: A Danish population-based cohort study (2019–2022)
Source: Heliyon. 2024 Jun 27;10(13):e33696. doi: 10.1016/j.heliyon.2024.e33696 (PMC11261871; doi:10.1016/j.heliyon.2024.e33696)
Supplement: Multimedia component 2 [file mmc2.docx]

| **Table S1.** Study population for the sensitivity analysis characteristics and 90-day mortality in patients with bacteremia, including only the first episode per patient. | | | |
| --- | --- | --- | --- |
| **Characteristic** | **Study population**^1^  n = 12,693 | **Dead at day 90**  n = 3,325 | **Alive at day 90**  n = 9,368 |
| **Sex** |  |  |  |
| Female | 5,644 (100%) | 1,437 (25%) | 4,207 (75%) |
| Male | 7,049 (100%) | 1,888 (27%) | 5,161 (73%) |
| **Age^2^** | 75 (65, 83) | 79 (72, 87) | 74 (62, 81) |
| **Origin of infection** |  |  |  |
| Community acquired | 6,830 (100%) | 1,309 (19%) | 5,521 (81%) |
| Healthcare associated | 2,898 (100%) | 908 (31%) | 1,990 (69%) |
| Nosocomial | 2,965 (100%) | 1,108 (37%) | 1,857 (63%) |
| **Charlson Comorbidity Index** |  |  |  |
| Low (0 points) | 3,542 (100%) | 504 (14%) | 3,038 (86%) |
| Medium (1–2 points) | 4,670 (100%) | 1,267 (27%) | 3,403 (73%) |
| High (>2 points) | 4,481 (100%) | 1,554 (35%) | 2,927 (65%) |
| **Pathogen** |  |  |  |
| *Escherichia coli* | 4,200 (100%) | 783 (19%) | 3,417 (81%) |
| *Enterococcus faecalis* | 310 (100%) | 86 (28%) | 224 (72%) |
| *Enterococcus faecium* | 346 (100%) | 162 (47%) | 184 (53%) |
| *Klebsiella pneumoniae* | 770 (100%) | 207 (27%) | 563 (73%) |
| *Pseudomonas aeruginosa* | 209 (100%) | 79 (38%) | 130 (62%) |
| *Staphylococcus aureus* | 1,651 (100%) | 546 (33%) | 1,105 (67%) |
| *Streptococcus pneumoniae* | 331 (100%) | 70 (21%) | 261 (79%) |
| *Streptococcus pyogenes* | 108 (100%) | 27 (25%) | 81 (75%) |
| Coagulase-negative staphylococci | 190 (100%) | 58 (31%) | 132 (69%) |
| *Candida* species | 220 (100%) | 125 (57%) | 95 (43%) |
| Polymicrobial | 1,072 (100%) | 367 (34%) | 705 (66%) |
| Other species | 3,286 (100%) | 815 (25%) | 2,471 (75%) |
| **Time period of infection** |  |  |  |
| February 01, 2019, to March 11 2020 | 4,791 (100%) | 1,258 (26%) | 3,533 (74%) |
| Covid-19 restriction period (March 12, 2020, to May 20, 2021) | 4,711 (100%) | 1,207 (26%) | 3,504 (74%) |
| May 21, 2021, to June 18 2021 | 288 (100%) | 72 (25%) | 216 (75%) |
| Strike period (June 19, 2021, to August 28, 2021) | 799 (100%) | 194 (24%) | 605 (76%) |
| August 29, 2021, to February 28 2022 | 2,104 (100%) | 594 (28%) | 1,510 (72%) |
| **Coinfection^3^ with SARS-CoV-2** | 576^4^ (100%) | 221 (38%) | 355 (62%) |
| (1) Number of bacteremia episodes (%) between February 01, 2019, to February 29, 2022, and mortality follow-up until May 29, 2022. (2) Median (inter quartile range). (3) Laboratory confirmed Covid-19 infection within +/- 30 days from the bacteremia episode. (4) Corresponding to SARS-CoV-2 co-infection in approximately 7.3% of all first episodes of bacteremia diagnosed between March 2020 (following the first confirmed Covid-19 case in Denmark on February 28, 2020) and February 2022. | | | |

| **Table S2.** Sensitivity analysis of the associations between Covid-19 restriction period, coinfection with SARS-CoV-2, strike period, and all-cause mortality in patients with bacteremia only including the first episode per patient. (12,693 cases) | | | | | | | | |
| --- | --- | --- | --- | --- | --- | --- | --- | --- |
| **Predictor variables** | **All-cause mortality** | **Model 1** | | **Model 2** | | **Model 3** | |  |
|  |  | HR (95% CI) | p-value | HR (95% CI) | p-value | HR (95% CI) | p-value |  |
| **Covid-19 restriction period^1^**  (ref. remaining period) | 0–30 days | 0.96  (0.88 to 1.04) | 0.29 | 0.93  (0.86 to 1.01) | 0.10 | 0.92  (0.85 to 1.01) | 0.065 |  |
|  | 31–90 days | 0.91  (0.79 to 1.04) | 0.15 | 0.90  (0.79 to 1.04) | 0.15 | 0.90  (0.78 to 1.03) | 0.12 |  |
|  | 0–90 days | 0.94  (0.88 to 1.01) | 0.10 | 0.92  (0.86 to 0.99 | 0.032 | 0.91  (0.85 to 0.99) | 0.019 |  |
| **Strike period^2^**  (ref. remaining period) | 0–30 days | 0.80  (0.68 to 0.95) | 0.009 | 0.85  (0.71 to 1.02) | 0.083 | 1.00  (0.82 to 1.23) | 0.96 |  |
|  | 31–90 days | 1.11  (0.88 to 1.40) | 0.39 | 1.04  (0.80 to 1.34) | 0.78 | 1.21  (0.92 to 1.59) | 0.17 |  |
|  | 0–90 days | 0.91  (0.79 to 1.06) | 0.23 | 0.91  (0.78 to 1.05) | 0.18 | 1.06  (0.90 to 1.26) | 0.47 |  |
| **Coinfection^3^**  **with SARS-CoV-2**  (ref. no coinfection) | 0–30 days | 1.60  (1.37 to 1.87) | <.001 | 1.60  (1.37 to 1.86) | <.001 | 1.30  (1.11 to 1.52) | <.001 |  |
|  | 31–90 days | 1.61  (1.22 to 2.10) | <.001 | 1.62  (1.23 to .2.12) | <.001 | 1.32  (1.00 to 1.74) | 0.047 |  |
|  | 0–90 days | 1.60  (1.40 to 1.83) | <.001 | 1.61  (1.41 to 1.84) | <.001 | 1.30  (1.13 to 1.50) | <.001 |  |
| HR = Hazard ratio; CI = Confidence interval. Model 1: Age adjusted HR for each predictor variable. Model 2: Adjusted for the predictor variables and age. Model 3: Full model adjusted for the predictor variables and all covariates (age, sex, month of bacteremia, acquisition of bacteremia [community acquired, hospital associated, or nosocomial], pathogen, and Charlson comorbidity index [categorized as low, medium or high index score]). (1) March 12, 2020, to May 20, 2021. (2) June 19, 2021, to August 28, 2021. Study period: February 01, 2019, to February 28, 2022, with follow-up for mortality until May 29, 2022. (3) Laboratory confirmed Covid-19 infection within +/- 30 days from the bacteremia episode. | | | | | | | | |
